# Island ancient genomes reveal dynamic populations interactions in the northern China

**DOI:** 10.3389/fmicb.2025.1584315

**Published:** 2025-04-16

**Authors:** Xu Zhang, Fan Zhang

**Affiliations:** ^1^Key Laboratory of Archaeological Sciences and Cultural Heritage, Chinese Academy of Social Sciences, Beijing, China; ^2^School of Archaeology and Museology, Sichuan University, Chengdu, China; ^3^Center for Archaeological Science, Sichuan University, Chengdu, China

**Keywords:** Tuoji Island, ancient DNA, Miaodao Archipelago, Liaodong Peninsula, Dakou site

## Abstract

The Longshan period (2500–1900 BC) was a transformative era in central China, marked by the emergence of complex social structures and early state formation. While human mobility likely played a role in these developments, the scale and nature of migration during this period remain poorly understood. Previous ancient DNA studies on Longshan culture populations have focused on individuals from inland Shandong, with no ancient DNA data available from island populations. In this study, we present the first ancient DNA analysis from individuals associated with the Longshan and subsequent Yueshi cultures on the Tuoji Island. Our findings indicate that, despite the widespread cultural influence of the inland Longshan culture in Shandong, the genetic ancestry of the Tuoji Island individuals primarily reflects connections to the preceding Dawenkou culture, with additional ancestry linked to the coastal regions of southern China. This suggests an earlier population movement into Tuoji Island before the Longshan period. However, during the Longshan period, the spread of Longshan cultural materials on Tuoji Island appears to represent the diffusion of ideas rather than significant population admixture from the inland. Additionally, our study shows genetic continuity of Longshan and Yueshi cultures in Tuoji Island highlighting the dynamic nature of coastal migration, as the Tuoji Island populations exhibit more genetic influence from coastal regions than from the inland. In contrast, inland populations during the Longshan period show no significant genetic influx from neighboring regions. This study not only advances our understanding of the prehistoric populations in Neolithic China but also provides new insights into patterns of migration and cultural exchange during this critical period.

## 1 Introduction

The Miaodao Archipelago, located between the two peninsulas of Jiaodong and Liaodong, occupies a key position in the study of the role of maritime pathways in prehistoric cultural exchange and human migration across East Asia. Made up of several islands only a few nautical miles apart, the archipelago is a natural bridge between the two peninsulas (Wu, 2021a). Due to this geographical closeness, vast maritime connections can be established, making the archipelago a central point in the early maritime system (Wu, 2019). Thereby, they acted as a bridge for human migration, and they also became the center for spreading agriculture, culture, and technology among the islands in the Miaodao Archipelago (Lv, 1989).

The Miaodao Archipelago was an active interaction center in the Neolithic period (Jiaxin et al., 1993), especially in the Longshan period (2500-1900 BCE). Through the archaeological work conducted on these islands, it is possible to observe a rich material culture of dolmens, stone tools, ceramics, and other artifacts, all of this within the framework of the exchange of practices and innovations (Xu, 2016). Both archaeological and linguistic evidence strongly suggest that the islands had long served as a conduit for the migration of people and agricultural practices, particularly rice farming, as well as the transmission of pottery design techniques and burial customs (Kazuo, 2019). These exchanges played an important role in the formation of prehistoric societies on the Jiaodong and Liaodong Peninsulas and represented long-distance interaction of culture and technology across regional units that contained a large geographical area (Shandong Peninsula, Korean Peninsula, and even the Japanese Archipelago). Perhaps the greatest cultural exchange was their involvement in the spread of rice agriculture (Sattar et al., 2010). The maritime routes were also pivotal in the diffusion of rice cultivation from the Yangtze River Basin, across the Shandong Peninsula to the Bohai Sea and into the Korean Peninsula and the Japanese Archipelago. The maritime pathways connecting the Miaodao Archipelago served as significant waypoints for the transfer and transmission of agriculture, including the development of the technologies around irrigation and agricultural farming tools (Wu, 2021b). These exchanges played a significant role not only in local economies but in shaping Neolithic societies in coastal areas. This role of the archipelago in mediating these agricultural and technological transfers to these more northeastern reaches of the East Asian continent highlights the importance of the archipelago in shaping the early agricultural landscape of East Asia. Recent years have witnessed new directions in the study of the human populations of the Miaodao Archipelago at the Longshan period through the archaeological attention (Zhang and Zhang, 2024).

Recent ancient DNA studies in the northern part of East Asia have shown suggested dynamic population movement with major archaeological cultural changes always combined with human population movements and admixtures (Mao et al., 2021; Ning et al., 2020; Robbeets, 2017; Wang C.-C. et al., 2021; Yang et al., 2020). Ancient DNA analyses from the middle and lower reaches of the Yellow River show that populations associated with the Middle Neolithic Dawenkou culture in Shandong province inherited not only the inhabitant hunter-gatherer ancestry but also involved genetic contribution from the Yangshao culture from the middle reaches of the Yellow River (Du et al., 2024; Fang et al., 2025; Liu et al., 2025). By the Late Neolithic the Longshan cultural individuals show continuous genetic contribution from the Yangshao cultural but an additional ancestry associated with populations from the Yangtze River Valley where rice was first domesticated was observed, showing that population from the southern part of China admixed into the populations in this region (Ning et al., 2020). From the Longshan culture until the Iron Age, populations in Central Plain show strong genetic continuity with limited or no genetic contribution from the surrounding regions (Ning et al., 2020). Compared with the lower reaches of the Yellow River, the middle reaches of the Yellow River show strong populations expansions with the Yangshao culture individuals contributed 60% of their ancestry to the Hongshan cultural individuals from the West Liao River Valley in Northeast China and this influence reaches nearly 100% during the Lower Xiajiadian period a culture subsequent to the Hongshan culture (Ning et al., 2020). Population dynamic expansion of the middle reaches of the Yellow River also left profound influence to the west and southwest of China, for example, they contributed around 90% of their genes to the Neolithic individuals from Sichuan and Yunnan as well as in the Tibetan Plateau (Tao et al., 2023).

Despite these insights, recent genomic studies from coastal Shandong have highlighted the region’s potential role as a migration corridor, with evidence of close genetic relationships between post-Yayoi populations from the Japanese Archipelago and coastal populations (Liu et al., 2025). However, all the ancient genomes published so far in China come from the continental mainland, and ancient genomes from the islands remain absent from the literature. This gap limits our understanding of prehistoric human migration across East Asia. To address this, we present ancient genomes from the Dakou site on Tuoji Island in the Miaodao Archipelago, providing new data to help elucidate migration patterns in this critical region.

## 2 Materials and methods

### 2.1 Archaeological context

**Figure 1:**
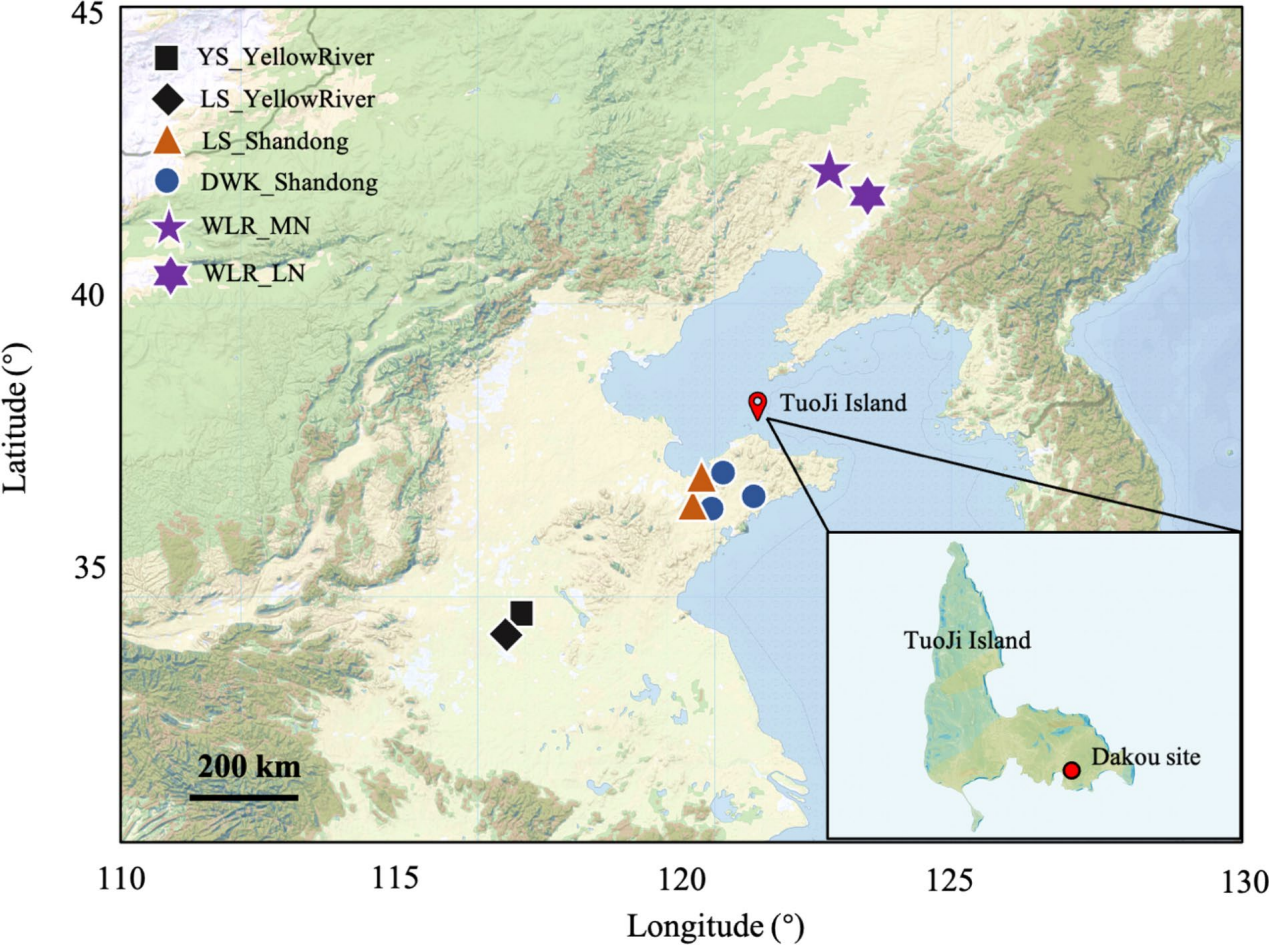
Geographic location of the Dakou archaeological site as well as published ancient DNA relevant to this study.

The Dakou site on Tuoji Island is located in Tuoji Town, Penglai District, Yantai City, Shandong Province, at the southern foot of Qiong Ren Ding Mountain, which rises to an elevation of 119 meters (Figure 1). In November and December of 1980, during the construction of water pipes by a local commune hospital, four tombs were discovered on the northern slope of the hospital. Archaeologists from the Archaeology Department of Peking University, along with local cultural heritage management departments, conducted a rescue excavation. Based on the artifacts unearthed, including eggshell pottery double-layer cups, the site was inferred to date to the Longshan culture period. In September 1982, to prevent further damage from the local hospital’s plans to build a wall on the site, archaeologists from the Institute of Archaeology of the Chinese Academy of Social Sciences, together with the Changdao County Museum, conducted a trial excavation. This excavation covered an area of 95 m^2^ at an elevation of 34 m, approximately 270 m from the coastline, uncovering two house sites, 22 tombs, nine animal pits, and ten fire trace.

Radiocarbon dating of the human skeletal remains recovered during the 1982 excavation indicated that the first phase of the site corresponds to the Longshan culture, dating from 2285 BCE to 1564 BCE, while the second phase corresponds to the Yueshi culture, dated from 2046 BCE to 1482 BCE (Wu and Shandong Team, Institute of Archaeology, Chinese Academy of Social Sciences, 1985). In total, 19 human skeletons were uncovered, with 10 from the Longshan culture and 9 from the Yueshi culture. The stratigraphy and artifacts, including pottery and bone tools, were used to differentiate the two cultural phases and support the chronological framework established by the radiocarbon dates. We collected teeth samples from 6 individuals for ancient DNA analyses.

### 2.2 Ancient DNA extraction, library preparation

We initially screened 6 ancient samples (Table 1) in dedicated clean facilities at the ancient DNA lab of Sichuan University, China, following established protocols for DNA extraction and library preparation (Dabney et al., 2013; Korlević et al., 2015). Prior to sampling, all skeletal elements were wiped with 5% bleach and irradiated with UV light for 30 min on each side. Teeth were drilled to obtain fine powder using a dental drill (Dremel, USA). Approximately 50 mg of powder from teeth was digested in 900 μL of 0.5 M EDTA (Sigma-Aldrich), 16.7 μL of Proteinase K (Sigma-Aldrich), and 83.3 μL of ddH_2_O (Thermo Fisher, USA) at 37°C for 20 h. After digestion, the supernatant was transferred to a MinElute silica spin column (QIAGEN, Germany), mixed with 13 mL of custom binding buffer [5 M guanidine hydrochloride (MW 95.53), 40% isopropanol, 90 mM sodium acetate (3 M), and 0.05% Tween-20], followed by two washes with PE buffer (80% ethanol). DNA was then eluted with 100 μL TET buffer (QIAGEN, Germany). We prepared a double-stranded dual-indexed library with a partial Uracil-DNA-glycosylase (UDG) treatment from a 20 μL aliquot of each DNA extract. Blunt-end repair of DNA fragments was performed by adding T4 Polynucleotide Kinase (0.5 U/μl; Thermo Fisher) and T4 DNA Polymerase (0.08 U; Thermo Fisher), incubating at 25°C for 15 min (Rohland et al., 2015). Repaired DNA fragments were purified using a standard MinElute purification step (QIAGEN, Germany), eluted in 18 μL TET buffer. Illumina adapters (0.25 μM adapter mix) were ligated to the blunt ends using 1X Quick Ligase (New England Biolabs, NEB) in a 40 μL total reaction volume, followed by another MinElute purification. The final fill-in step was performed by adding 1X isothermal buffer, 0.4 U/μL Bst-polymerase (NEB), and 250 μM dNTP mix (Thermo Fisher), incubating at 37°C for 30 min and 80°C for 20 min. Libraries were indexed using uniquely combined double indices. Indexed products were purified using AMPure XP beads (Beckman Coulter Ltd), and library concentrations were quantified using a Qubit 2.0 fluorometer (Thermo Fisher). Finally, libraries were sequenced on an Illumina HiSeq X10 instrument (Annoroad Company, China) using a 150-bp paired-end sequencing design. Sequence reads were demultiplexed by allowing one mismatch in each of the two 8-bp index sequences.

**Table 1:** Summary of genetic sequencing of the Dakou individuals in this study.

ID	Group	nr.Total reads	len.trimmed	nr.1240 K	Mt haplogroup	Culture
M3	TuoJi_G1	65,451,012	45.8072	89,798	D5c2	Yueshi
M2	TuoJi_G2	71,305,516	47.9545	31,201	B4a1 + 16,311	Yueshi
M8	TuoJi_G2	62,939,050	47.1162	63,798	B4b1c	Longshan
M12	TuoJi_lc	64,522,408	46.1201	2,792	–	Longshan
M6	TuoJi_lc	66,137,660	53.0378	8,492	D4b1a	Yueshi
M17	TuoJi_lc	52,560,034	44.9928	984	D5b1	Yueshi

### 2.3 Sequencing and data processing

Sequence reads were demultiplexed by allowing one mismatch in each of the two 8-bp index sequences. Illumina sequencing adapters were clipped using AdapterRemoval v2.3.3 (Schubert et al., 2016). Merged reads were then mapped to the human reference genome (hs37d5; GRCh37 with decoy sequences) using BWA v0.7.17 (Li and Durbin, 2010). PCR duplicates were removed with DeDup v0.12.8 (Peltzer et al., 2016). To minimize the impact of postmortem DNA damage on genotyping, we generated additional “trimmed” BAM files by soft-masking the first and last 10 base pairs of each read using the trimbam function from bamUtils v1.0.15 (Jun et al., 2015), based on the DNA misincorporation pattern observed in each library. For SNP genotyping in the 1240 K panel (Haak et al., 2015; Mathieson et al., 2015), a single high-quality base (Phred-scaled base quality score of 30 or higher) was randomly sampled as pseudodiploid genotypes using the pileupCaller program.^1^ For C/T and G/A SNPs, we used the trimmed BAM files, while for other SNPs, untrimmed BAM files were employed.

### 2.4 Genetic sexing and uniparental haplogroup assignment

We determined the molecular sex of our ancient samples by comparing the ratio of X and Y chromosome coverage to that of the autosomes (Fu et al., 2016). mtDNA consensus sequences were generated for each individual using Geneious v11.1.3 software (Kearse et al., 2012), and haplogroups were assigned using HaploGrep3 (Weissensteiner et al., 2016).

### 2.5 Population structure analysis

We performed principal component analysis (PCA) using smartpca v16000 (Patterson et al., 2006), incorporating a reference set of 2,077 present-day Eurasian individuals from the “HumanOrigins” dataset and a subset of 266 East Asian individuals from the “1240 k-Illumina” dataset. PCA was conducted with the options “lsqproject: YES” and “shrinkmode: YES.” Additionally, we carried out unsupervised admixture analysis with ADMIXTURE v1.3.0 (Alexander et al., 2009). Genetic markers with a minor allele frequency less than 1% were excluded, and linkage disequilibrium was pruned using the “--indep-pairwise 200 25 0.2” option in PLINK v1.90 (Chang et al., 2015). To assess genetic relationships between the target population and Eurasian populations since their divergence from an African outgroup, outgroup *f3* statistics were calculated (Supplementary Table S9). Additionally, *f4* statistics were computed using the ‘f4mode: YES’ function in the ADMIXTOOLS package. Both *f3* and *f4* statistics were calculated employing qp3Pop v435 and qpDstat v755 in the ADMIXTOOLS package (Patterson et al., 2012).

### 2.6 Admixture modeling with qpAdm

We performed admixture modeling for our ancient northern China populations using the qpWave/qpAdm framework (qpWave v410 and qpAdm v810) (Haak et al., 2015). Nine populations from the “HumanOrigins” and “1240 k-Illumina” datasets were used as outgroups (OG1): Mbuti, Natufian, Onge (Onge.DG in the “1240 k-Illumina” panel), Iran_N, Villabruna, Mixe (Mixe.DG in the “1240 k-Illumina” panel), Ami (Ami.DG in the “1240 k-Illumina” panel), Nganasan, and Itelmen (Itelmen.DG in the “1240 k-Illumina” panel). These populations represent a broad genetic diversity, including an African outgroup (Mbuti), early Holocene Levantine hunter-gatherers (Natufian), Andamanese islanders (Onge), early Neolithic Iranians from the Tepe Ganj Dareh site (Iran_N), late Pleistocene European hunter-gatherers (Villabruna), Central Native Americans (Mixe), indigenous Samoyedic people from the Taymyr Peninsula (Nganasan), and an ethnic group native to the Kamchatka Peninsula (Itelmen). Given that some ancient northern Chinese populations exhibit increased genetic affinity with Nganasan and Itelmen, we also modeled these populations by excluding Nganasan and Itelmen from the outgroup set.

## 3 Results

### 3.1 Physical anthropological analyses of the Dakou individuals

Cranial matrix measurements were meticulously conducted on six well-preserved adult individuals—three males and three females—from the Late Longshan culture at the Dakou site. The males were identified from M12, M20, and M22, while the females were identified from M8, M15, and M16 (Zhang et al., 2023). These findings reveal that, despite the considerable consistency in craniofacial morphological characteristics among the ancient inhabitants during the Dawenkou culture period in Shandong, the evolution into the Longshan culture period brings about a fascinating increase in complexity and nuance in cranial morphology. The human skeletal remains excavated from the Longshan phase at the Dakou site, along with those from the Dinggong site in central-northern Shandong and the Chengziya site in northern Shandong, display the key craniofacial morphological features characteristic of the Dawenkou culture population. Importantly, these remains indicate a noteworthy shift away from a brachycephalic (round skull) shape, transitioning instead to a mesocephalic (medium-width skull) feature, while the tendency towards a broad nose becomes more prominent. Conversely, the skeletal remains from the Xiwusi site in southwestern Shandong, although also exhibiting this inclination towards a broad nose, continue to show a brachycephalic (round skull) cranial index (Supplementary Tables S1–S5). This variance may be attributed to the differing timelines at which each site adopted the Longshan culture, coupled with the unique local iterations of the Longshan culture to which they belonged. Understanding these morphological changes not only sheds light on the evolution of cranial features but also enhances our comprehension of cultural adaptation in ancient societies.

M8 and M12 also underwent ancient DNA testing. M8, a 30-year-old female, has a relatively complete skull with a narrow, dolichocranial shape, underdeveloped occipital features, and broad facial traits; the pelvic bones are incomplete, and the bones were covered with conch shells and pebbles during excavation, indicating a specific burial practice. M12, a male aged 30–35, exhibits an oval skull with moderately developed brow ridges and a square chin, showing traits similar to modern Chinese populations. His craniometric analysis suggests a dolichocranial, high cranial type with a narrow form, and missing portions of the pelvic and sacral bones; stones found pressed against the bones again indicate a specific mortuary practice.

### 3.2 Ancient genome data production from the Dakou site

We initially screened 6 ancient individuals from Dakou site using shallow shotgun sequencing, with one Illumina sequencing library per individual (Table 1). The results show that Dakou samples were not well preserved and with only 3 individuals exceed endogenous human DNA over 1%. The authenticity of the genome data was verified through multiple measures. All samples exhibited postmortem chemical damage typical of ancient DNA (Supplementary Figure 1) and ensuring that the estimated modern human DNA contamination was below 5% (Supplementary Table S6). Haploid genotypes were produced for 593,124 autosomal SNPs from the Affymetrix “HumanOrigins” platform. These genotypes were merged with published ancient genomic data and present-day individuals from the “HumanOrigins” and “1240 k” datasets. For group-based analyses, we primarily categorized the ancient individuals by their date, geographic region, archaeological context, and genetic profile.

### 3.3 Genetic structure of the Dakou individuals

**Figure 2:**
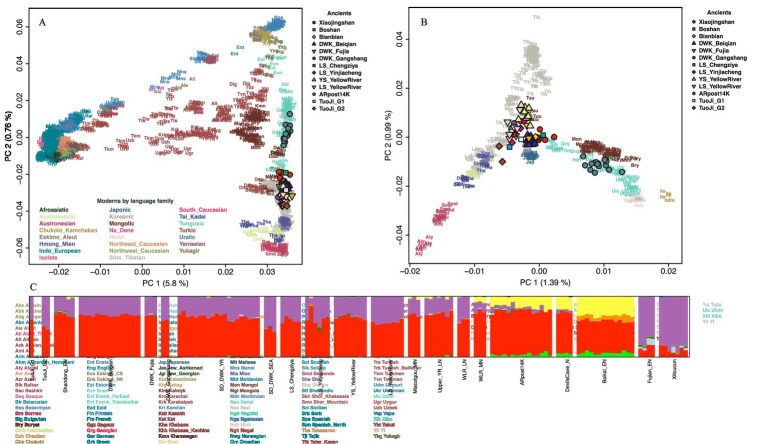
A summary of the genetic profiles of ancient Dakou and present-day East Asian populations. **(A)** The first two principal components constructed from 2077 present-day Eurasians; the ancient individuals are projected onto the first two PCs. Color-filled shapes represent ancient individuals. **(B)** The first two principal components calculated from present-day individuals from 18 East Asian populations. **(C)** ADMIXTURE results for the “HumanOrigins” dataset at K = 4. Only the East Asian populations are plotted.

To gain a comprehensive understanding of the genetic profile of ancient Shandong populations, we first projected our samples onto the top two principal components (PC1 vs. PC2) of present-day Eurasian population genetic variation. As expected, all three individuals from the Dakou site fell within the genetic variation of present-day East Asians, as well as published ancient genomes from the region, particularly along the PC1 axis (Figure 2A). This suggests that the genetic makeup of the Dakou individuals aligns closely with other East Asian populations, providing a foundation for further investigation of their specific genetic affinities. To explore variation within East Asians more thoroughly, we expanded the analysis to include a panel of 18 present-day East Asian populations. The first two principal components successfully distinguished distinct groups, including Tungusic-speaking populations (e.g., Oroqen, Hezhen, Xibo), Tibetans, and populations from Southern China and Southeast Asia (Figure 2B). The same pattern was also observed in unsupervise Admixture analysis that Dakou individuals from the Tuoji island shares the same genetic structure with the both ancient and present-day populations in East Asia (Figure 2C; Supplementary Figure 5) and share the most genetic drift with Neolithic individuals from Shandong (Supplementary Figure 2).

Notably, the Dakou individuals were separated into two distinct clusters based on their genetic affinities (Figure 2). One cluster, labeled Tuoji_G1, comprises individual M3 and aligns genetically with populations from the Longshan culture as well as the Dawenkou culture of the Yellow River Valley. This cluster demonstrates a clear connection to the broader genetic landscape of northern China. In contrast, the second cluster, includes two individuals, M2 and M8, from the Yueshi and Longshan period, respectively. Symmetry test suggest that M2 and M8 are cladal to each other we thus group the two individuals into a single group, Tuoji_G2. Compared to Tuoji_G1, Tuoji_G2 shows a notable shift towards populations from southern China, indicating a possible influence from this region during the Longshan period. Despite these regional shifts, outgroup f3-statistics analyses reveal that both Tuoji_G1 and Tuoji_G2 share the highest genetic affinity with each other when compared to other ancient and present-day populations from East Asia (Supplementary Figure 2; Supplementary Table S7). This genetic relationship underscores the interconnectedness of the Dakou individuals, suggesting a common genetic origin or significant gene flow between these two groups. Furthermore, the highest shared genetic affinity was observed with ancient individuals from the Shandong province, which geographically neighbors the Tuoji Island. This proximity indicates that continental genetic influences from the surrounding regions, particularly Shandong, played a significant role in shaping the genetic structure of the Dakou individuals during the Longshan period. These findings provide important insights into the genetic landscape of ancient populations in Shandong and surrounding areas, highlighting the complex interplay of regional genetic influences that contributed to the genetic diversity of the Longshan culture.

### 3.4 Distinct genetic structure compared with contemporaneous individuals from mainland

**Figure 3:**
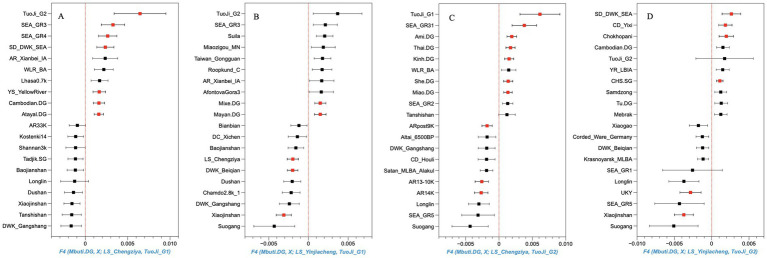
Symmetry tests for Dakou individuals from Tuoji Island. We present the 10 most positive (upper side) and 10 most negative (lower side) *f4* (Mbuti.DG, X; Shandong Longshan, Tuoji_G1/G2) statistics across 334 world-wide populations. Horizontal bars represent the point estimate ±1 s.e.m., with the s.e.m. estimated using 5 cM block jackknifing. The |Z| values greater than 3 are marked in red. Specifically: **(A)**. F4 (Mbuti.DG, X; LS_Chengziya, Tuoji_G1); **(B)**. F4 (Mbuti.DG, X; LS_Yinjiacheng, Tuoji_G1); **(C)**. F4 (Mbuti.DG, X; LS_Chengziya, Tuoji_G2); **(D)**. F4 (Mbuti.DG, X; LS_Yinjiacheng, Tuoji_G2).

Outgroup-f3 statistics analyses show that despite the genetic substructure of the Dakou individuals, they share the highest genetic affinity with preceding and contemporaneous individuals from inland Shandong including those associated with the Dawenkou culture as well as the Longshan culture (Supplementary Figure 2). However, the Dakou individuals were genetically different from the inland Longshan cultural individuals showing a shift to populations from southern China in PCA. This signal was further confirmed by the symmetry test of the form *f4*(Mbuti, X; LS_Chengziya/LS_Yinjiacheng, Tuoji_G1/ Tuoji_G2), where X represent various ancient and present-day populations from East Asia, and LS_Chengziya and LS_Yinjiacheng represent inland Longshan culture populations from Shandong province. As expected, both Tuoji_G1 and Tuoji_G2 show significant genetic affinity with populations from southern China (Figure 3; Supplementary Figures 3, 4), and southeast Asia, showing that compared with the Longshan cultural individuals from inland Shandong, the island Longshan individuals show further genetic affinity with populations from southern China, suggesting extra or a different wave of population admixture into the Tuoji island. Dakou individuals can be best modeled by two-way admixture as evidenced by the qpAdm analyses. Both Tuoji_G1 and Tuoji_G2 were modeled with the Dawenkou individuals from inland Shandong, and another ancestry associated with individuals from the coastal region of southern China (e.g., Liangdao individuals from Fujian province), and the majority ancestry from inland Shandong (80%) and the remaining 20% from southern China (Supplementary Table S8). This result shows that genetic influence from inland Shandong preceded the Longshan period, and during the Longshan period, the expansion of the Longshan culture into Tuoji Island was merely a dispersal of ideas and did not involve large-scale population movement.

## 4 Discussion

The Longshan period (c. 2500–1900 BC) was a transformative era in central China, marked by the emergence of complex social structures and early state formation. This period saw the spread of material culture and ideological practices across vast networks, contributing to the development of urban centers by the late third millennium BC (Chang, 1989; Yan, 1981; Zhao, 2000). While human mobility likely played a role in these changes, the scale and nature of migration remain poorly understood. Shandong, the center of Longshan culture, is a mountainous region with settlements found on surrounding plains. Key sites include Chengziya, Dinggong, Tianwang, and Bianxianwang, with Chengziya being the largest (20 ha). Liangchengzhen (273 ha) and Yaowangcheng (368 ha) are the largest sites in Shandong, located near the southeast coast, surrounded by smaller, economically integrated settlements, suggesting they were political centers of competing polities. These sites produced pottery, stone tools, textiles, and prestige items made from jade and metal. Agricultural practices included rice, millet, and wheat, with foxtail millet being the most widely grown crop, though primarily used for animal fodder. The Dakou site, located on the Tuoji Island, is associated with the Longshan culture based on both its material culture and its strategic geographic position (Zhang and Zhang, 2024). This provides a unique opportunity to examine the role of coastal migrations and interactions with inland populations during the Neolithic period through the lens of ancient DNA analysis.

Genetic analysis of the Dakou individuals reveals a strong genetic affinity with populations from inland Shandong (Fang et al., 2025; Liu et al., 2025; Yang et al., 2020), specifically those linked to the Dawenkou and Longshan cultures. This genetic evidence suggests that Tuoji Island not only received material cultural influences from the mainland but was also subject to population movements. However, despite the temporal overlap with the Longshan period, our qpAdm analyses indicate that the Longshan populations from Central Plain do not provide a good model for the genetic composition of the Dakou individuals. Instead, the genetic structure of the Dakou population more closely resembles that of the preceding Dawenkou culture, particularly the Fujia archaeological site (Du et al., 2024), suggesting an early migration of populations into the Tuoji Island during the Neolithic. This population movement appears to have occurred before the Longshan period, with the Longshan influence likely limited to the diffusion of ideas and material culture rather than large-scale population migration. Interestingly, while the Dakou individuals share a strong genetic link with the Dawenkou populations from Fujia, they cannot be modeled solely by this ancestry. A better fit is achieved when another genetic component is introduced, specifically from coastal populations of southern China, such as those from the Fujian region (Yang et al., 2020). This genetic contribution from the southern coast indicates a complex pattern of population dynamics, where not only inland groups but also coastal populations influenced the genetic makeup of the Tuoji Island inhabitants. This genetic evidence starkly contrasts with the inland Shandong populations associated with the Longshan culture, whose genetic structure appears predominantly derived from the preceding Dawenkou culture, with no significant genetic input from southern China (Wang T. et al., 2021). Instead, the Longshan period individuals in inland Shandong seem to have also inherited genetic contributions from the Yangshao culture, which predates the Longshan period. This highlights a distinct pattern of genetic continuity and the lack of substantial genetic influx from southern China during the inland Longshan period, emphasizing the regional specificity of population movements. The presence of genetic contributions from the coastal regions in the Dakou population suggests a strong dynamic of population movement along the southeastern coast of China during the Neolithic. This pattern indicates that coastal migration routes were significant during the period, facilitating cultural and genetic exchanges between the inland and coastal regions.

These results offer new evidence for Neolithic population dynamics that challenges the long-held notion that the inland migrations and cultural expansion associated with the Longshan period were the primary processes occurring in Neolithic China. Rather, the findings emphasize the importance of coastal areas in the population genetic structure of the Longshan culture and characterize migration in this process as more complicated. Dakou also had higher genetic diversity than groups in the two previous sites, indicating the culmination of both appealing expansion and dynamic interaction along the coast. It also has implications for understanding the development of social networks, the spread of modes of agriculture, and the role of maritime ways in prehistoric China.

## Data Availability

The raw DNA data presented in this study have been deposited in the NCBI Sequence Read Archive (SRA) under BioProject PRJNA1131741 (available at https://www.ncbi.nlm.nih.gov/bioproject/PRJNA1131741/).

## Footnotes

^1^ https://github.com/stschiff/sequenceTools
