# Supplementary material for: Island ancient genomes reveal dynamic populations interactions in the northern China
Source: Front Microbiol. 2025 Apr 16;16:1584315. doi: 10.3389/fmicb.2025.1584315 (PMC12040904; doi:10.3389/fmicb.2025.1584315)
Supplement: Supplementary file 1 [file Data_Sheet_1.docx]

Supplementary Material

## Supplementary Figures


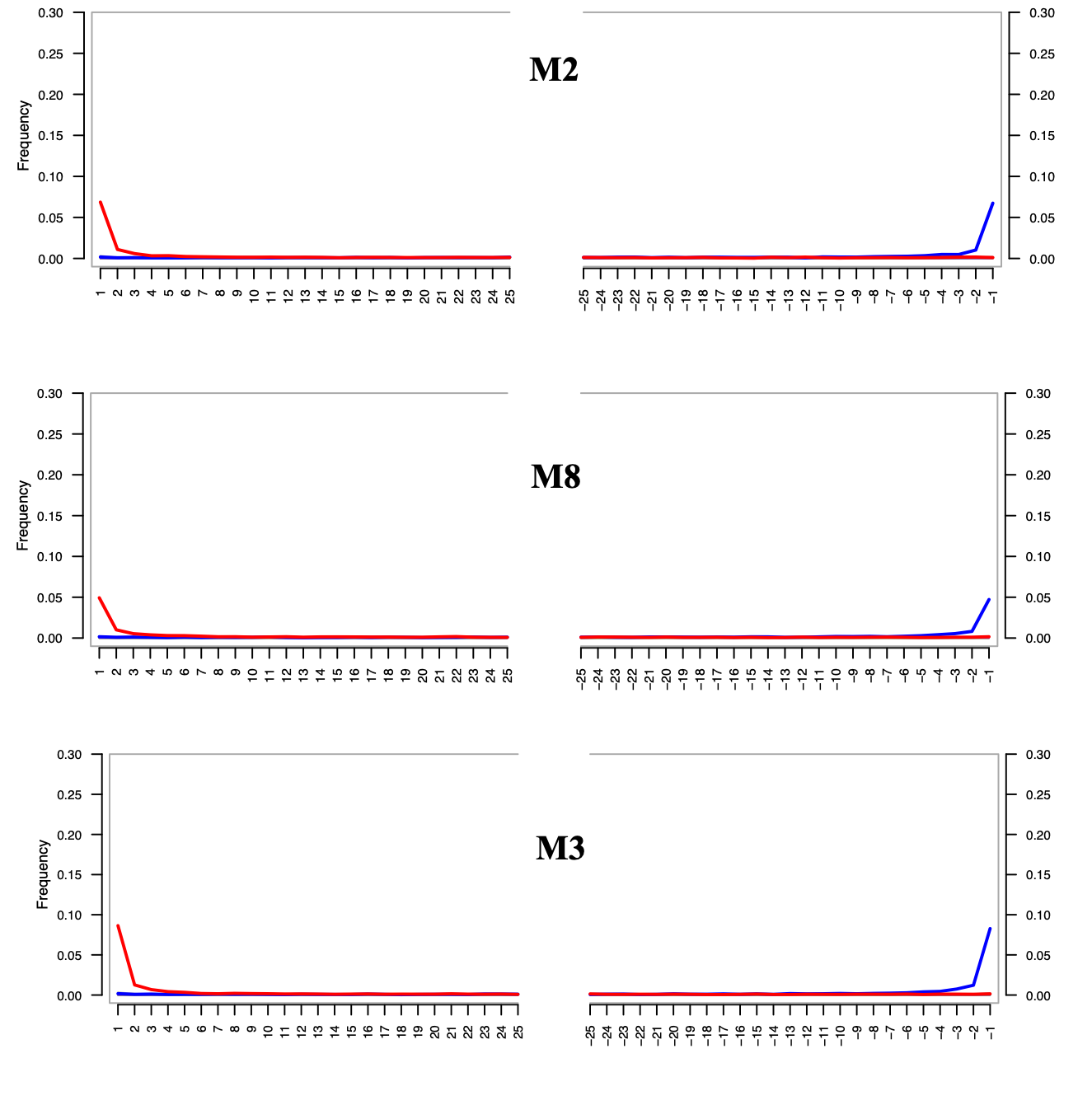


**Supplementary Figure 1.** **DNA damage plots for 3 ancient samples from Dakou.** Curves on the left side represent C->T misincorporations at the 5’ end and those on the right side represent G->A misincorporations at the 3’ end.


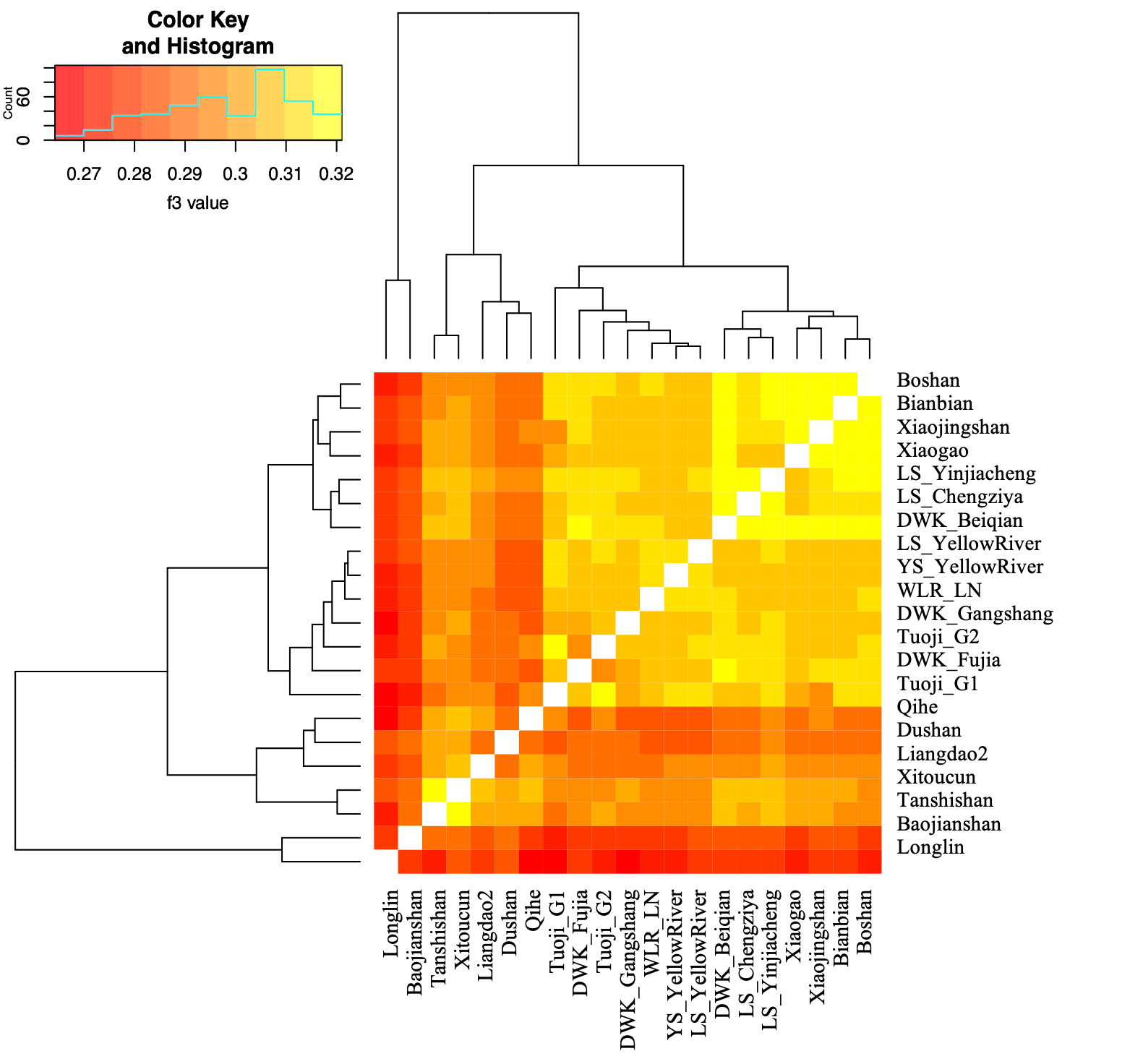


**Supplementary Figure 2.** **A heatmap summary of the outgroup *f*3-statistics for pairs of ancient and present-day East Asian populations.**


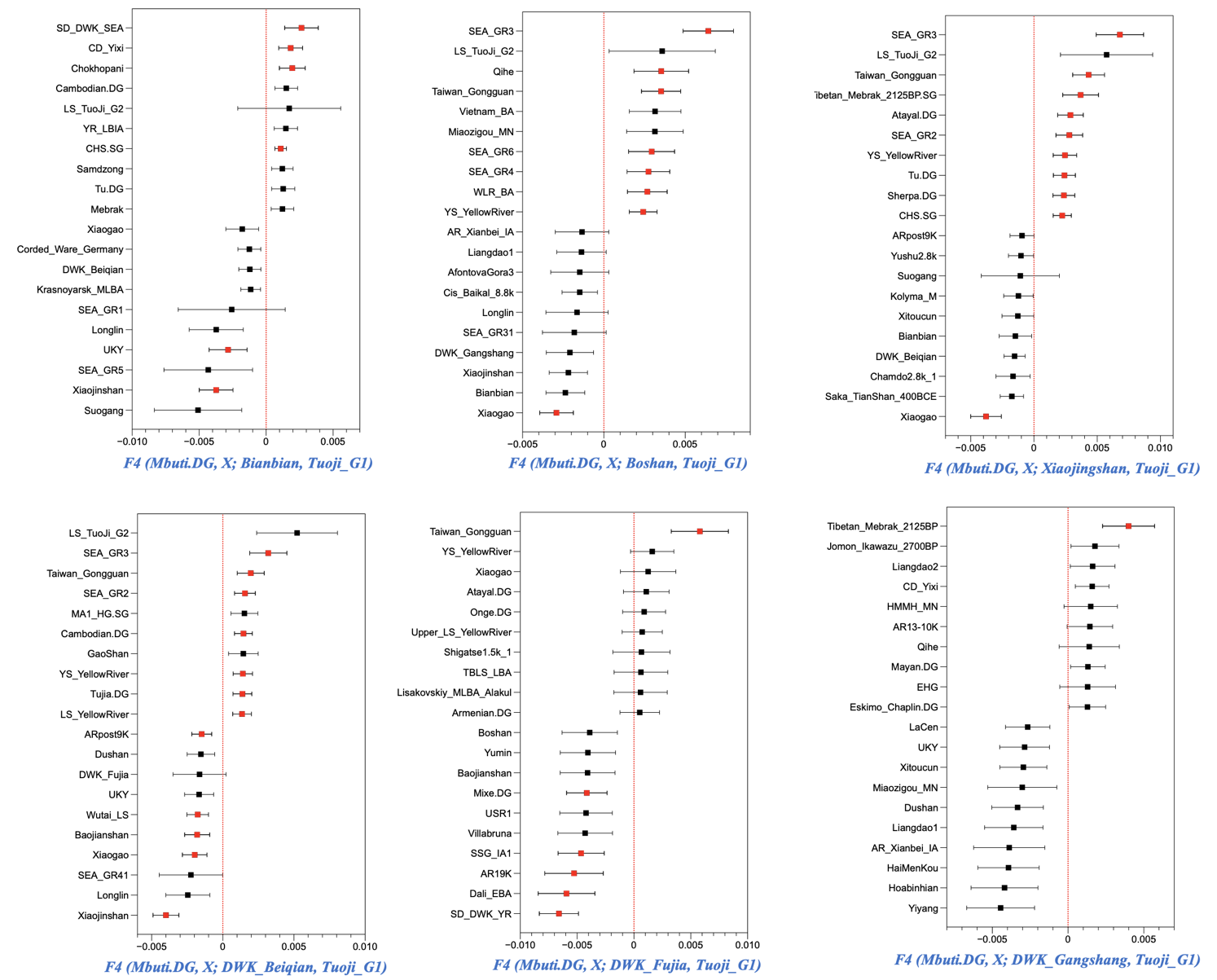


**Supplementary Figure 3.** **The genetic difference between Tuoji_G1 and Neolithic Shandong.** We present 10 most positive (upper side) and 10 most negative (lower side) *f4*(Mbuti.DG, X; Shandong Dawenkou, Tuoji_G1) statistics across 334 world-wide populations. Horizontal bars represent the point estimate ± 1 s.e.m. The s.e.m. are estimated using 5 cM block jackknifing. The |Z| values greater than 3 are marked in red.


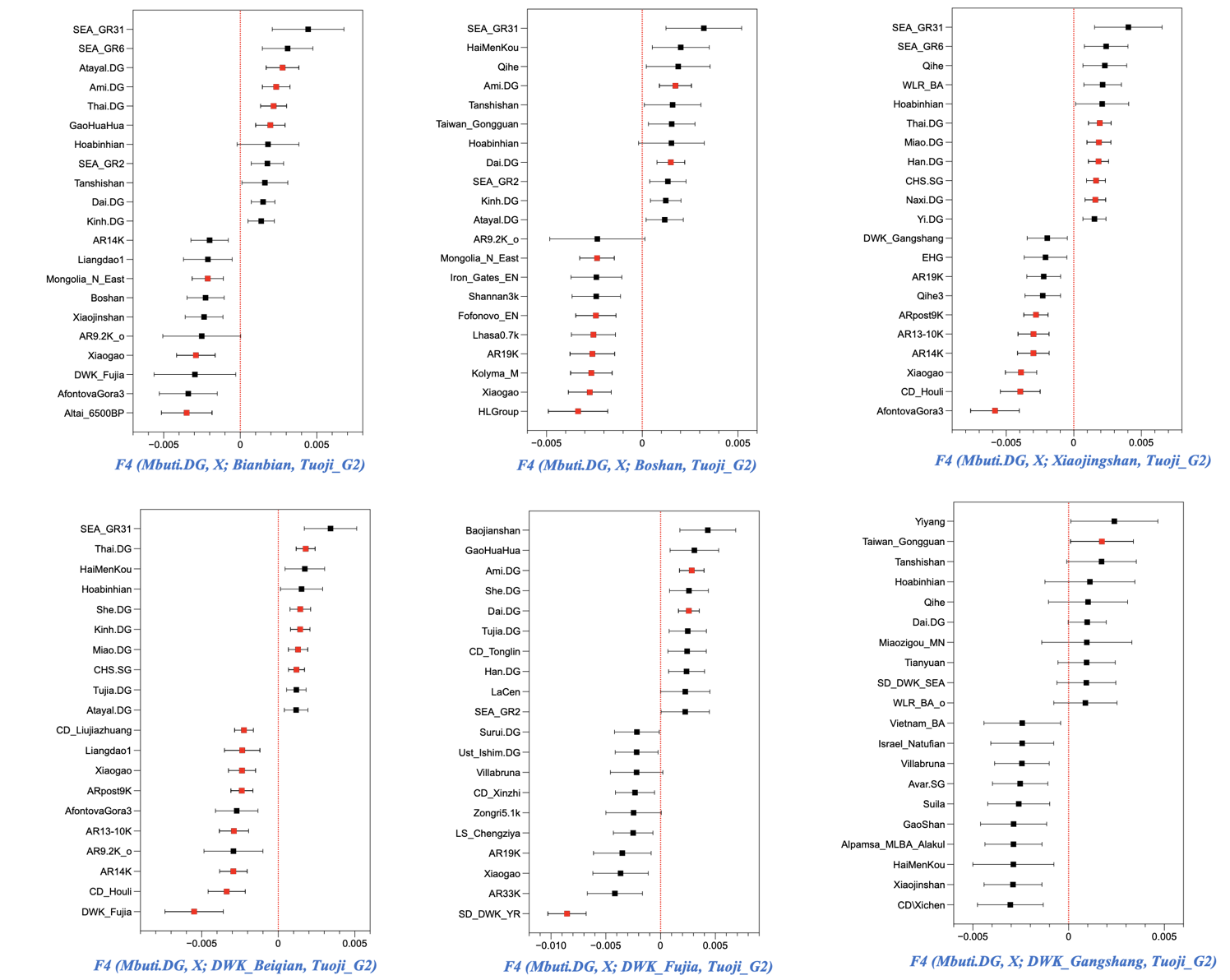


**Supplementary Figure 4.** **The genetic difference between Tuoji_G2 and Neolithic Shandong.** We present 10 most positive (upper side) and 10 most negative (lower side) *f4*(Mbuti, X; Shandong Dawenkou, Tuoji_G2) statistics across 334 world-wide populations. Horizontal bars represent the point estimate ± 1 s.e.m. The s.e.m. are estimated using 5 cM block jackknifing. The |Z| values greater than 3 are marked in red.

**
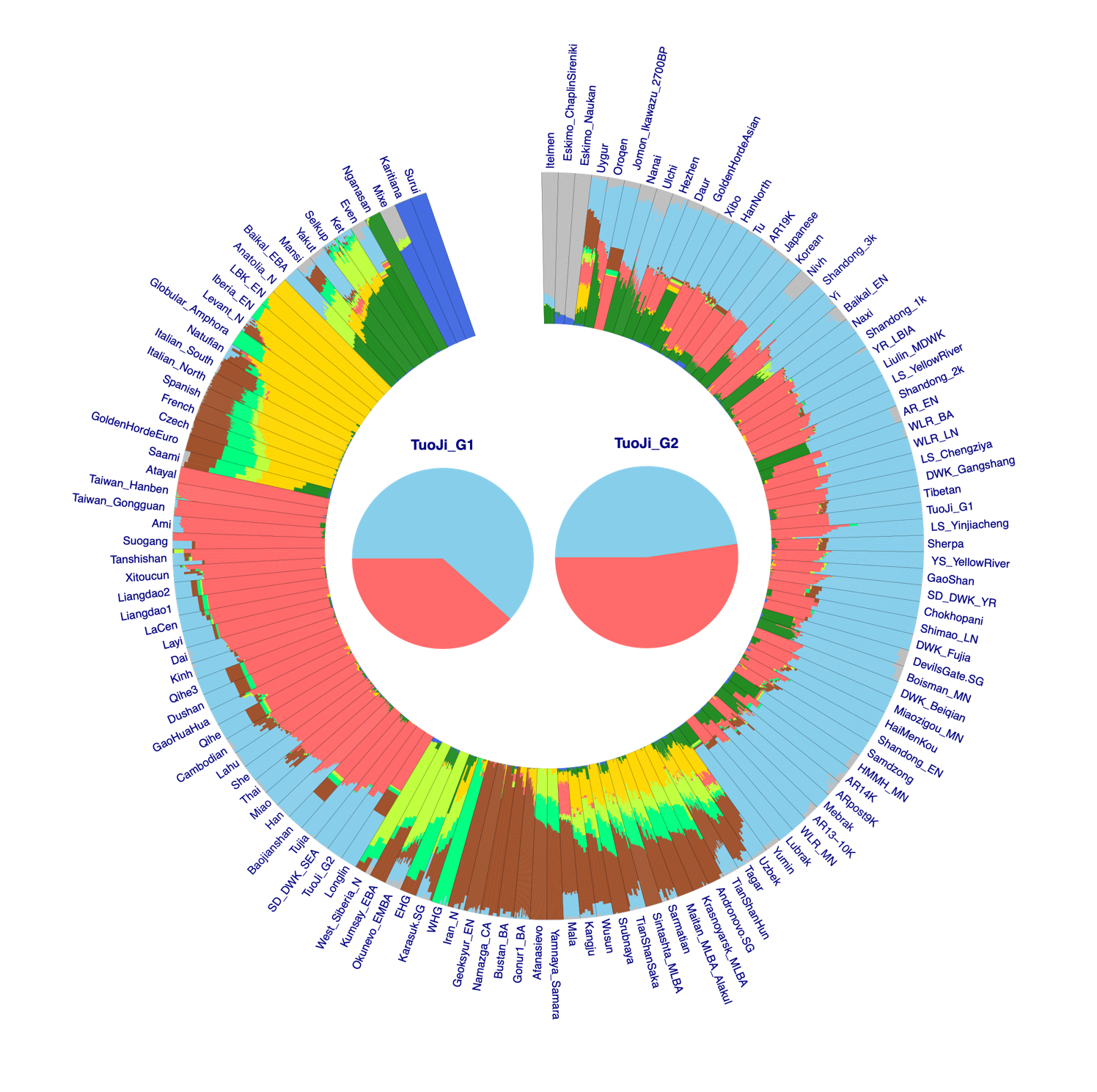
**

**Supplementary Figure 5. ADMIXTURE results for K=9**. We include previously published ancient and present-day populations across Eurasia.
